# Comparison between a linear versus a macrocyclic contrast agent for whole body MR angiography in a clinical routine setting

**DOI:** 10.1186/1532-429X-10-63

**Published:** 2008-12-30

**Authors:** Achim Seeger, Ulrich Kramer, Michael Fenchel, Florian Grimm, Christiane Bretschneider, Jörg Döring, Bernhard Klumpp, Gunnar Tepe, Kilian Rittig, Peter R Seidensticker, Claus D Claussen, Stephan Miller

**Affiliations:** 1 Department of Diagnostic and Interventional Radiology, Eberhard-Karls-University Tuebingen, Hoppe-Seyler-Str. 3, 72076 Tuebingen, Germany; 2 Department of Internal Medicine IV, Eberhard-Karls-University Tuebingen, Otfried-Müller-Str. 10, 72076 Tuebingen, Germany; 3 Bayer Schering Pharma AG, 13342 Berlin, Germany

## Abstract

**Background:**

Previous experiences of whole body MR angiography are predominantly available in linear 0.5 M gadolinium-containing contrast agents. The aim of this study was to compare image quality on a four-point scale (range 1–4) and diagnostic accuracy of a 1.0 M macrocyclic contrast agent (gadobutrol, n = 80 patients) with a 0.5 M linear contrast agent (gadopentetate dimeglumine, n = 85 patients) on a 1.5 T whole body MR system. Digital subtraction angiography served as standard of reference.

**Results:**

All examinations yielded diagnostic image quality. There was no significant difference in image quality (3.76 ± 0.3 versus 3.78 ± 0.3, p = n.s.) and diagnostic accuracy observed. Sensitivity and specificity of the detection of hemodynamically relevant stenoses was 93%/95% in the gadopentetate dimeglumine group and 94%/94% in the gadobutrol group, respectively.

**Conclusion:**

The high diagnostic accuracy of gadobutrol in the clinical routine setting is of high interest as medical authorities (e.g. the European Agency for the Evaluation of Medicinal Products) recommend macrocyclic contrast agents especially to be used in patients with renal failure or dialysis.

## Background

Atherosclerosis is one of the leading challenges of health care in developed countries. Due to the complex pathophysiology and systemic nature of the cardiovascular system [1] there is a need for accurate assessment of the manifestations in order to offer best treatment, including surgical and percutaneous catheter-based interventions as well as pharmacological therapy. In this setting, Cardiovascular Magnetic Resonance (CMR) is increasingly used [2-4].

Current imaging strategies have been developed with linear 0.5 M contrast agents, for example gadopentetate dimeglumine [2,4,5]. In the context of nephrogenic systemic fibrosis (NSF) macrocyclic contrast agents are recommended to be used for contrast-enhanced MRI in patients at risk [6-8]. The very high complex stability of macrocyclic contrast agents decrease the risk to release gadolinium ions in vivo. Gadobutrol (Gadovist™, Bayer Schering Pharma, Germany) became the first macrocyclic 1.0 M contrast agent to receive approval from the European Union for contrast-enhanced MR angiography. Due to its more compact bolus profile gadobutrol is well suited for dynamic imaging (for example perfusion imaging). As it is increasingly used in clinical routine for CMR imaging, the diagnostic value of gadobutrol is of high interest.

Using gadobutrol requires modifications with regard to volume properties, circulation time and flow rates as gadobutrol has a higher viscosity and is higher concentrated compared to gadopentetate dimeglumine. According to our knowledge, no comparison of the diagnostic performance of gadobutrol to the standard gadopentetate dimeglumine in whole body MR-Angiographie (WBMRA) in a large patient group using the identical sequence protocol has been reported. Aim of this study was to compare image quality and diagnostic accuracy in WBMRA in a clinical routine setting in 165 patients (gadopentetate dimeglumine n = 85, gadobutrol n = 80). Conventional X-ray angiography served as standard of reference and was available in the area of suspected pathology based upon the symptoms.

## Methods

### Study population

In this retrospective analysis we included 165 patients with clinical evidence of peripheral artery disease (PAD, Fontaine stage IIb-IV) who underwent DSA and WBMRA within 14 days from January 2004 to June 2007. The patients were examined either using the 0.5 M gadopentetate dimeglumine (Magnevist™, Bayer Schering Pharma, Germany) or the 1.0 M gadobutrol (Gadovist™, Bayer Schering Pharma, Germany) which was mainly used after the knowledge that NSF might be associated with the release of gadolinium ions in vivo.

The mean age in the gadopentetate dimeglumine group (n = 85, 66 male, 19 female) was 66 ± 10.1 years and in the gadobutrol group (n = 80, 58 male, 22 female) 67 ± 11.9 years. A glomerular filtration rate < 30 mg/dl according to the Cockcroft-Gault formula was present in 5 patients (gadopentetate dimeglumine group n = 2, gadobutrol group n = 3).

### MR Imaging protocol

**Figure 1:**
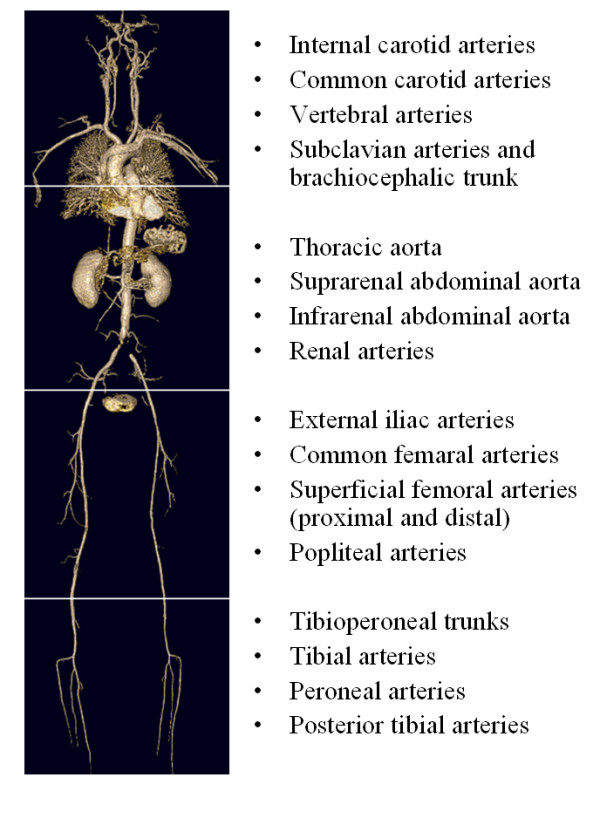
**Acquisition and Analysis**. the four overlapping fields of view (head and neck; thorax and abdomen; upper leg; lower leg) that were used for acquisition. On the right side, the 31 segments for image analysis are labelled. The figure shows a 3D reconstruction of the vascular tree of an 50 year old man with stent in the common iliac arteries. Contrast agent for WBMRA in this patient was gadobutrol.

All examinations were performed on a 1.5 T whole body MR-scanner (Magnetom Avanto, Siemens Medical Solutions, Erlangen, Germany). For signal reception, surface coils were used for all body regions. Four overlapping fields of view of 500 mm were used for covering the arterial vascular system (see table 1 for imaging parameters and Fig. 1). Scout images were obtained from all stations. Unenhanced and enhanced images were acquired from the abdominal region and the upper and lower leg area by using 3D FLASH sequences, the time to move the table between the stations was about 2–3 seconds. 15 minutes after the first contrast medium application unenhanced and enhanced images of the neck and head were measured.

**Table 1:** Imaging parameters.

	**TR (ms)**	**TE (ms)**	**FA (°)**	**FOV (mm^2^)**	**Matrix**	**Slice (mm)**	**GRAPPA**	**TA (sec)**	**Bandwidth (Hz/Px)**
**Head and Neck**	2.85	1.68	25	344 × 500	264 × 512	1.6	x2	17	650
**Abdomen**	3.11	1.14	25	375 × 500	230 × 512	1.5	x2	13	420
**Upper Leg**	3.46	1.21	25	375 × 500	230 × 512	1.5	x2	12	360
**Lower Leg**	3.46	1.21	25	375 × 500	230 × 512	1.3	x2	13	360

Table 1 shows the imaging parameters of WBMRA of the 4 overlapping segments that are shown in Fig.1. Generalized autocalibrating partially parallel acquisition (GRAPPA) in phase encoding direction was used. Scan duration times for a single station were 12 – 17 seconds (time of acquisition, TA).

### Contrast media and injection

In total 0.25 mmol/kg body weight (bw) contrast media was injected. The scan delay was determined with a sagittal test bolus section angled to the course of the aorta. An automated injector (Spectris, Medrad) was used in all examinations. Gadobutrol was administered at a flow rate of 1.2 ml/s (depending on the patients weight 10–13 ml for the abdominal region and the legs resulting in a typical bolus duration of 8–11 seconds and 5–7 ml for the head and neck), Gadopentetate dimeglumine was injected with biphasic flow for the abdomen and legs (0.15 mmol/kg bw @ 2 ml/s followed by 0.03 mmol/kg bw @ 1 ml/s resulting in a typical bolus duration of 10–16 seconds). All contrast injections were followed immediately by a saline flush (25 ml @ 1.0 ml/s).

A timing bolus was used (gadopentetate dimeglumine 2 ml or gadobutrol 1 ml) followed by 25 ml saline flush and image delay was calculated according to the following formula:

T_cir _- T_k _+ 4 sec (where T_cir _is the circulation time and T_k _is the time to the k-space center line).

### Conventional Digital Subtraction Angiography (DSA)

DSA of the area of suspected pathology based upon the symptoms (either comprising the abdominal aorta, the renal and pelvic arteries and the arteries of the lower extremities or in some cases only the symptomatic lower extremity) was performed with a standard angiographic unit (Axiom Artis TA; Siemens Medical Systems) using a 4 French straight catheter transfemorally and 20 ml of contrast agent (Ultravist 370™, Bayer Schering Pharma, Germany) was administered at each station. As required, examinations were supplemented with acquisition of one or more oblique views of the arteries.

### Image analysis

Baseline images were subtracted from the contrast-enhanced images for each station and maximum intensity projection reconstruction images were performed. All WBMRA images were assessed by two readers consensus reading. The maximum intensity projection reconstructions and the contrast enhanced source images were used for image analysis on a segment-by-segment basis. DSA images were evaluated by an experienced radiologist who had 8 years of experience in diagnostic and interventional angiographic procedures.

For image evaluation the vascular system was classified into a total of 31 arterial segments (see Fig.1). The readers were blinded to clinical symptoms, contrast agent and DSA. A four-level system rate for image quality of each arterial segment was used (1 = non-diagnostic images, 2 = poor image quality, significant blurring/artefacts, diagnosis suspected but not established, 3 = good quality with definite diagnosis, minimal blurring/artefacts, 4 = sharply defined borders, excellent quality image information). The images were evaluated for presence of venous superimposition that hampered image analysis and required a multiplanar reconstruction of the contrast-enhanced data sets.

Each vascular segment was assessed for the presence of hemodynamically relevant stenosis (which was defined as luminal narrowing of 70–99%) or occlusion.

MRA and DSA were compared in all segments for which data from both modalities were available.

### Statistical analysis

Continuous data are presented as mean ± standard deviation. Quantitative variables were tested for statistical significance by using a Student t-test. Statistical significance was defined by p < 0.05. All p-value were two tailed.

The results of WBMRA and DSA were compared in every available segment. Sensitivity, specificity, positive and negative predictive value and accuracy for the detection of hemodynamically relevant stenosis as well as Cohen κ value agreement were calculated.

## Results

### Qualitative analysis

All examinations (gadopentetate dimeglumine n = 85, gadobutrol n = 80) yielded diagnostic image quality. A total of 5115 vascular segments (31 segments per patient in 165 patients) were evaluated for the presence of vascular disease and image quality. The mean rating was 3.77 ± 0.3, there was no significant difference between the image quality rating of gadopentetate dimeglumine (3.76 ± 0.3) compared to gadobutrol (3.78 ± 0.3; p = n.s.).

Subject motion was the most common reason for reduced image quality in 12 patients (gadopentetate dimeglumine n = 5, gadobutrol n = 7). Venous superimposition in the lower leg that hampered the diagnostic assessment was evident in 10 patients (gadopentetate dimeglumine n = 6, gadobutrol n = 4).

### DSA findings

**Figure 2:**
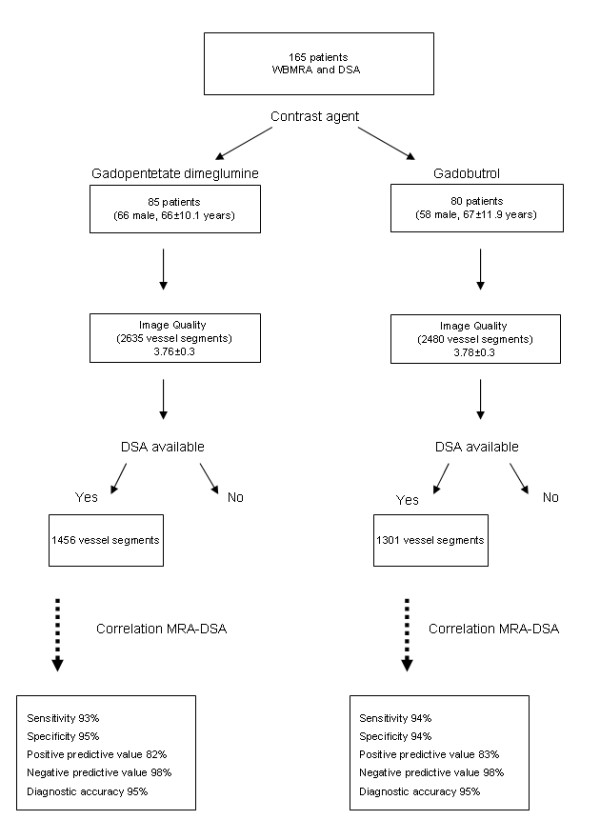
**Study Population and Results**. the flow diagram of the study population and the results of the correlation between DSA and WBMRA.

DSA of the symptomatic region was available in 2757 segments (1456 segments in the gadopentetate dimeglumine group and 1301 segments in the gadobutrol patients, see Fig.2).

In 2153 segments (1164 gadopentetate dimeglumine, 989 gadobutrol) no stenosis was seen, 249 segments (106 gadopentetate dimeglumine and 143 gadobutrol) showed a stenosis > 70% and 355 segments showed a vascular occlusion (186 gadopentetate dimeglumine and 169 gadobutrol).

### Correlation WBMRA to DSA

**Figure 3:**
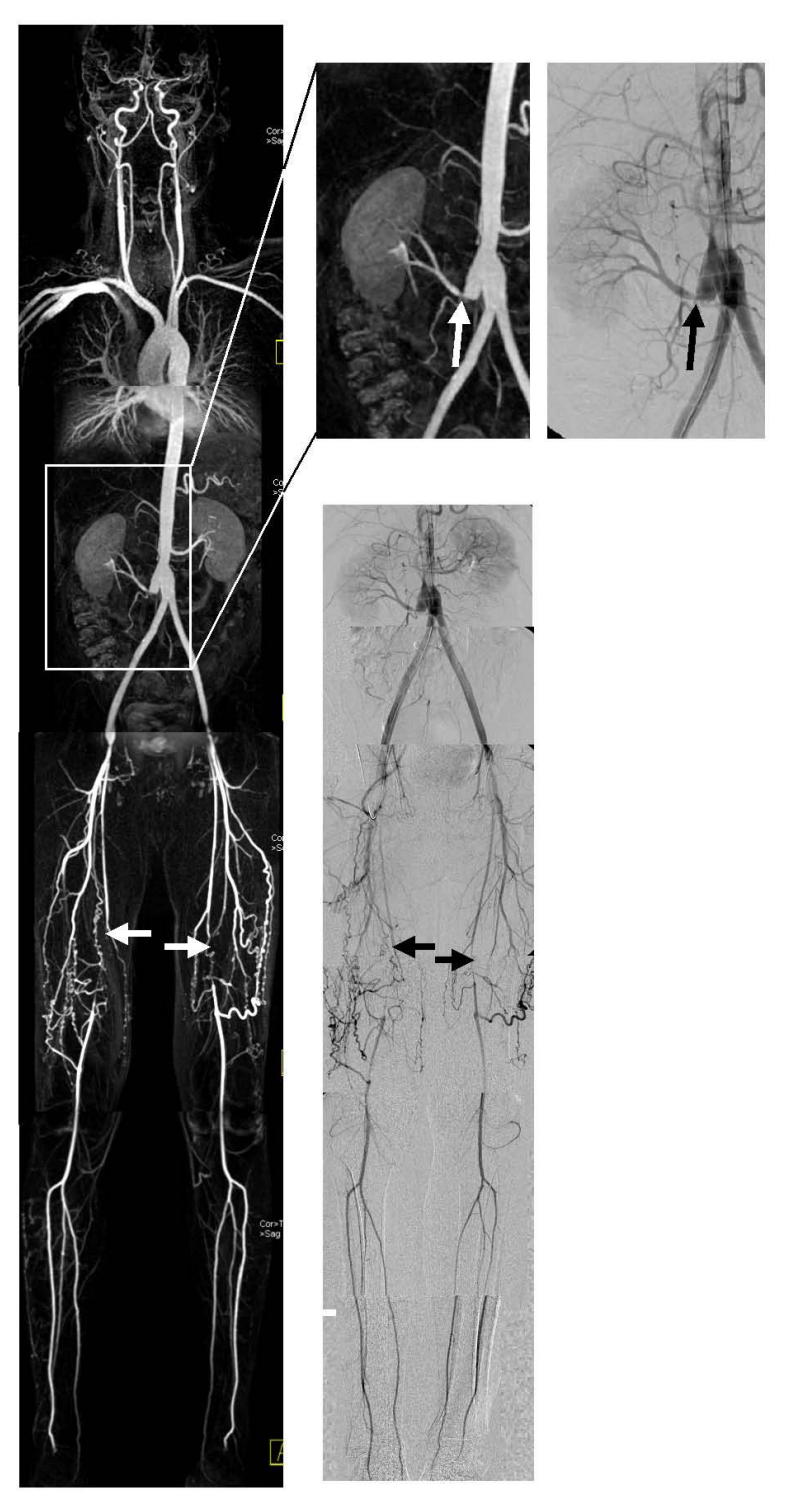
**Example gadopentetate dimeglumine**. 41 year old man with peripheral arterial occlusive disease stage Fontaine IIb (pain in both legs) and Y-prothesis. The left side shows the WBMRA (maximum intensity projection), on the right side the DSA. Both modalities show the occlusion of the superficial femoral arteries (arrows) and a stenosis of the right renal artery (arrows in the expanded images).

**Figure 4:**
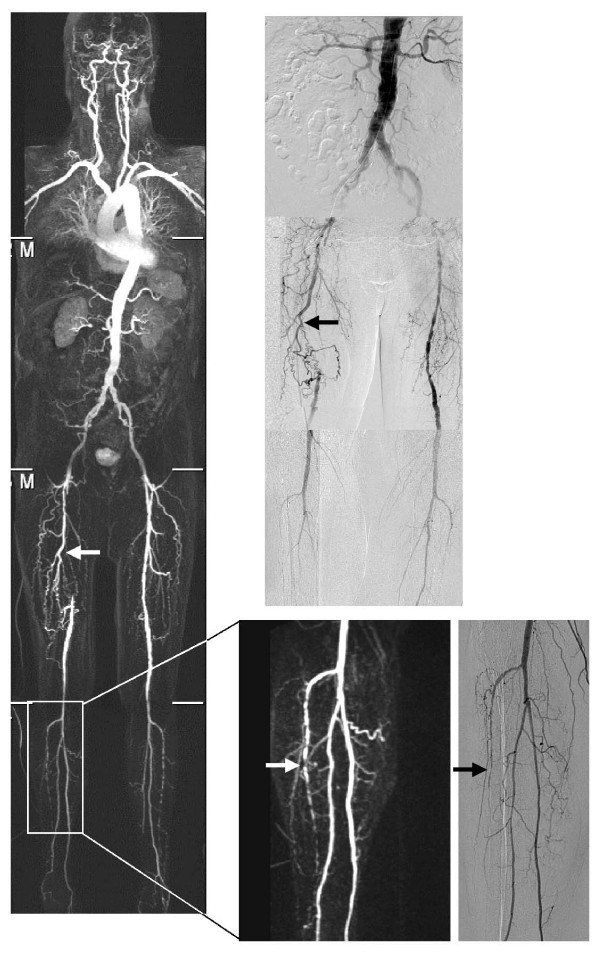
**Example gadobutrol**. 79 year old man with peripheral arterial occlusive disease stage Fontaine IIb (pain in the right leg) and stent in the right renal artery. The left side shows the WBMRA (maximum intensity projection), on the right side the DSA. Both modalities show the occlusion of the right superficial femoral artery (arrows) and the accurate correlation, for example the lesions in the right anterior tibial artery (arrows in the expanded images).

Fig.3 and Fig.4 show examples of the correlation between a maximum intensity projection WBMRA and DSA in both contrast agents.

In the gadopentetate dimeglumine group, 1369 of 1456 segments were correctly diagnosed (1100 without stenosis, 84 relevant stenoses and 175 vascular occlusions). WBMRA resulted in 16 false-negative vessel segments and 47 false-positive segments, 7 segments were overestimated and 6 segments underestimated.

In the gadobutrol group, 1212 of 1301 segments were correctly diagnosed (932 without stenosis, 122 relevant stenoses and 158 vascular occlusions). WBMRA resulted in 15 false-negative segments and 46 false-positive segments, 6 vessel segments were overestimated and 6 segments underestimated. While overall sensitivity/specificity for the detection of stenoses or occlusions in the gadopentetate dimeglumine group was 93%/95% it was 94%/94% using gadobutrol, respectively. Diagnostic accuracy was 95% for both contrast agents. Overall Kappa value agreement between WBMRA and DSA was 0.83 for gadopentetate dimeglumine as well as gadobutrol (see Fig.2).

Table 2 shows sensitivities, specificities, negative and positive predictive values and diagnostic accuracies in all segments for both study groups. In all vascular segments WBMRA showed good correlation to DSA, even in the lower leg regions there was high diagnostic accuracy in depiction of hemodynamically relevant stenoses. There were no statistically significant differences in the diagnostic performance between the study groups.

**Table 2:** Result correlation WBMRA-DSA

	Gadopentetate dimeglumine					Gadobutrol				
	Sensitivity	Specificity	NPV	PPV	Accuracy	Sensitivity	Specificity	NPV	PPV	Accuracy
Suprarenal aorta	-	100	100	-	100	100	100	100	100	100

Infrarenal aorta	100	100	100	100	100	100	100	100	100	100

Right renal artery	100	98	100	75	98	100	97	100	83	98

Left renal artery	100	100	100	100	100	100	100	100	100	100

Right common iliac artery	86	96	98	75	95	100	97	100	92	98

Left common iliac artery	100	98	100	86	98	94	100	97	100	98

Right external iliac artery	100	98	100	86	98	100	93	100	63	96

Left external iliac artery	100	98	100	89	98	100	100	100	100	100

Right common femoral artery	71	100	96	100	97	80	98	98	80	96

Left common femoral artery	80	98	98	80	97	88	96	98	78	95

Right SFA (proximal)	100	91	100	79	93	90	98	95	95	97

Left SFA (proximal)	100	94	100	88	97	96	97	97	96	97

Right SFA (distal)	87	95	92	91	93	97	93	97	94	95

Left SFA (distal)	89	95	93	92	94	100	94	100	94	97

Right popliteal artery	100	96	100	75	97	70	96	94	78	91

Left popliteal artery	93	98	98	93	97	83	98	96	91	97

Right tibioperoneal trunk	86	96	98	75	95	100	96	100	75	96

Left tibioperoneal trunk	91	93	98	71	92	100	98	100	88	98

Right ant. tibial artery	94	94	94	94	94	94	82	97	68	87

Left ant. tibial artery	95	87	98	76	89	94	88	97	75	90

Right peroneal artery	93	88	97	74	93	83	84	95	59	88

Left peroneal artery	94	83	98	68	89	81	86	92	68	88

Right posterior tibial artery	95	95	97	91	95	96	84	96	82	93

Left posterior tibial artery	93	89	94	87	91	96	80	97	76	88

Table 2 shows the result of the WBMRA of both groups (gadopentetate dimeglumine and gadobutrol) in correlation to DSA as standard of reference in all available arterial segments.

## Discussion

Atherosclerosis is a systemic disease that may involve the entire arterial system in man. Knowledge of coexisting lesions enables the appropriate treatment in a systemic context. In this setting WBMRA is increasingly used for vascular staging with high diagnostic accuracy compared to DSA as reference standard and its clinical impact on patient management has been shown in previous studies [3,9].

Because of the doubled gadolinium concentration of gadobutrol injection protocols like flow rate and injection volume have to be adapted to adjust the vascular enhancement to the acquisition window. While gadobutrol has been used in earlier studies with a rolling system (AngioSURF™, System for Unlimited Rolling Fields-of-View, MR-Innovation, Essen, Germany) [9,10] the published data using whole body scanners (TIM™, Total Imaging Matrix, Siemens, Germany) have been performed using 0.5 M extracellular gadolinium chelates like gadopentetate dimeglumine [5,11,12]. In this study the injection protocol of both gadopentetate dimeglumine and gadobutrol was optimised according to the experience of our institution. The mean gadobutrol injection rate in a multicenter study [13] was in correspondence to our protocol 1.2 ml/s and a high diagnostic accuracy for MRA of the body using versus DSA has been proved. The reported agreement rate of Schaefer et al. was 96.6% in the clinical evaluation and 86.6–90.2% in the independent blinded reader evaluation, but no comparison to a standard 0.5 M contrast agent has been performed in this study.

The published data on the potential benefits of gadobutrol to 0.5 M Gd-chelates are controversial. Goyen et al. reported an improved delineation of blood vessels in WBMRA [9] in a small patient group (3 volunteers, 10 patients) as well as an improved delineation of small vessels in the pelvic vascular territory [14]. Benefits in first-pass T2*-weighted cerebral perfusion have also been reported [15] while other studies showed no advantage of Gadobutrol for CE-MRA of the lung, abdomen as well as first-pass cardiac and pulmonary perfusion [16-18].

The purpose of our study was to compare the diagnostic performance of the 0.5 M gadopentetate dimeglumine and the 1.0 M gadobutrol in whole body MRA in a large patient group suffering from PAD using identical sequence parameters.

We found good to excellent image quality in both, the 0.5 M and the 1.0 M contrast agent. No significant difference in image quality between the two study groups was detected. Limitations in image quality were caused by subject motion of venous superimposition without significant differences in the two groups. Venous contamination could potentially be reduced by the use of thigh compression [19], nevertheless due to the rare appearance it is not routinely performed in our institution. An alternative approach to increase the image quality is the application of blood pool contrast agents like gadofosveset (Vasovist™, Bayer Schering Pharma, Germany). However the diagnostic benefit of blood pool contrast agents in WBMRA is still under investigation.

In comparison to conventional X-ray angiography, severe vascular occlusive disease was correctly diagnosed in an overall sensitivity/specificity of 93/95% (gadopentetate dimeglumine) and 94/94% (gadobutrol), respectively. Diagnostic accuracy (95%) and Kappa value agreement (0.83) was identically for both study groups. The values are close to those reported in the literature. Overestimation of stenoses was more frequently found than underestimation and is a known limitation of MRA [20]. Although i.a. DSA is accepted as the reference standard, discrepancies between WBMRA and DSA can be due to errors in either modality. The three-dimensional nature of WBMRA images potentially allows superior depiction of eccentric stenoses compared with the two-dimensional DSA.

As the needed dose for WBMRA is higher than for a single station MRA, a well tolerated contrast agent that allows high image quality should be chosen. Gadobutrol is known to be well tolerated [21,22] and in the context of NSF it is less likely prone to release Gadolinium ions in vivo. Due to the high co-morbidity of peripheral arterial disease (PAD) and renal failure, contrast-enhanced MR angiography (CE-MRA) is frequently indicated in this patient group [23] and gadobutrol might be increasingly used in risk patients.

New developments in sequence technique, parallel acquisition and fast-gradient technology allow rapid scan times to avoid venous enhancement and to achieve higher spatial resolution. In this setting, gadobutrol has the potential to enable a sharper bolus peak and therefore higher intravascular gadolinium concentration and may compensate the diminished SNR due to smaller voxel size.

A limitation is the fact that the comparison of the two study groups was based on qualitative parameters and the measurement of quantitative date like signal-to-noise ratio and contrast-to-noise ratio would be desirable. However applying parallel imaging techniques comes along with the problem that quantitative analysis require a complex correction [24] and is not relevant for the clinical routine use.

## Conclusion

We found a very good agreement between DSA and WBMRA in both contrast agents. WBMRA using the 1.0 M gadobutrol allows injection of less contrast agent volume and achieves comparable diagnostic accuracies to reported results of gadopentetate dimeglumine.

## Abbreviations

bw: body weight; DSA: digital subtraction angiography; GRAPPA: generalized autocalibrating partially parallel acquisition; FA: flip angle; FLASH: fast low angle shot; FOV: filed of view; NoS: number of slices; NPV: negative predictive value; PPV: positive predictive value; SL: slice thickness; TA: time of acquisition; TE: echo time; TR: repetition time; WBMRA: whole body MR-Angiography.

## Competing interests

PRS is employee of Bayer-Schering Pharma GmbH, Berlin, Germany.

## Authors' contributions

AS prepared the study design and drafted the manuscript, FG, CB and JD performed the MRI, UK, KR and BK performed image reading and participated in the coordination of the study. MF participated in the design of the study and helped to draft the manuscript and to perform the statistical analysis. PRS participated in the design of the study and helped to draft the manuscript. SM, UK and CDC participated in the study design and coordination and supervised the studies. GT, UK and SM performed the DSA. SM conceived the study design. All authors read and revised the article and approved the final manuscript.
